# Prevalence of Thyroid Dysfunction in Patients With Type 2 Diabetes Mellitus and Its Impact on Glycemic Control

**DOI:** 10.7759/cureus.113707

**Published:** 2026-07-31

**Authors:** Javaria Ashraf, Umar Ejaz, Ahmed Jamal Chaudhary, Nusratullah Khan, Sara Khan, Razwan Ashraf, Syed Muhammad Abdullah, Rizwan Ali

**Affiliations:** 1 Medicine, Northampton General Hospital, Northampton, GBR; 2 Medicine, Lahore General Hospital, Lahore, PAK; 3 Internal Medicine, DMC Sinai-Grace Hospital, Detroit, USA; 4 Biochemistry, Mekran Medical College Turbat, Turbat, PAK; 5 Pathology, CMH Institute of Medical Sciences Bahawalpur, Bahawalpur, PAK; 6 Medicine, Aziz Bhatti Shaheed Teaching Hospital, Gujrat, PAK; 7 Cardiothoracic Surgery, Tabba Heart Institute, Karachi, PAK; 8 Plastic and Cosmetic Surgery, Tongji Hospital, School of Medicine, Tongji University, Shanghai, CHN; 9 Institute of Aesthetic Plastic Surgery and Medicine, School of Medicine, Tongji University, Shanghai, CHN

**Keywords:** glycemic control, hba1c, hypothyroidism, thyroid dysfunction, type 2 diabetes mellitus

## Abstract

Background

Type 2 diabetes mellitus (T2DM) and thyroid dysfunction are common endocrine disorders. Thyroid hormones play an important role in glucose metabolism, insulin secretion, and insulin sensitivity. Disturbances in thyroid function may worsen glycemic control and increase the risk of diabetic complications. This study aimed to determine the prevalence of thyroid dysfunction in patients with T2DM and evaluate its impact on glycemic control.

Methods

This cross-sectional study was conducted at Nawaz Sharif Medical College/Aziz Bhatti Shaheed Teaching Hospital, Gujrat, Pakistan, from January to June 2025. A total of 260 patients with T2DM were enrolled through consecutive sampling. After exclusion of incomplete records, 252 patients were included in the final analysis. Demographic and clinical data were collected through interviews and medical records. Fasting blood glucose, glycated hemoglobin (HbA1c), thyroid-stimulating hormone (TSH), free triiodothyronine (FT3), and free thyroxine (FT4) were measured. Data were analyzed using IBM SPSS Statistics for Windows, Version 26 (Released 2019; IBM Corp., Armonk, New York).

Results

Among 252 participants, 68 (27.0%) had thyroid dysfunction while 184 (73.0%) were euthyroid. Subclinical hypothyroidism was the most common abnormality, affecting 15.1% of patients. Patients with thyroid dysfunction had significantly higher fasting blood glucose levels (189.7 ± 49.6 vs. 161.4 ± 42.8 mg/dL, p<0.001) and HbA1c levels (9.1 ± 1.7 vs. 7.8 ± 1.4%, p<0.001) compared to euthyroid patients. Poor glycemic control (HbA1c ≥7%) was more common in patients with thyroid dysfunction (83.8% vs. 58.7%, p<0.001). Multivariable logistic regression showed that thyroid dysfunction was independently associated with poor glycemic control (adjusted OR=2.74, 95% CI: 1.41-5.33, p=0.003).

Conclusion

Thyroid dysfunction is common among patients with T2DM and is associated with poor glycemic control. Routine thyroid screening may help identify high-risk patients and improve diabetes management.

## Introduction

Type 2 diabetes mellitus (T2DM) is one of the most common chronic diseases worldwide. It is a major public health problem and is associated with significant morbidity and mortality [1,2]. Pakistan is among the countries with a high burden of diabetes. Poor glycemic control can lead to serious complications, including cardiovascular disease, nephropathy, neuropathy, and retinopathy [3,4]. Therefore, identification of factors that affect glucose control is important for better patient management.

The thyroid gland is an important part of the endocrine system. It produces hormones that regulate metabolism, energy utilization, growth, and many physiological functions of the body. Thyroid hormones also influence glucose production, insulin secretion, and insulin sensitivity. Any disturbance in thyroid function may affect carbohydrate metabolism and worsen diabetes control [5].

Diabetes mellitus and thyroid dysfunction frequently occur together. Previous studies have reported that thyroid disorders are more common in patients with T2DM than in the general population [6]. Reported prevalence rates vary among different populations, but most studies have shown a prevalence ranging from 12% to 30%. Subclinical hypothyroidism is the most frequently reported thyroid abnormality in diabetic patients [7].

The relationship between thyroid dysfunction and glycemic control is complex [8]. Hypothyroidism may reduce glucose utilization by peripheral tissues and increase insulin resistance. Hyperthyroidism may increase intestinal glucose absorption and hepatic glucose production [9]. Both conditions can adversely affect blood glucose levels. Several studies have shown that thyroid dysfunction is associated with higher fasting blood glucose and HbA1c levels in patients with T2DM [10].

Despite the growing burden of diabetes in Pakistan, local data regarding thyroid dysfunction among diabetic patients remain limited, especially in the Balochistan region. Early detection of thyroid abnormalities may help improve metabolic control and reduce the risk of complications. Therefore, the present study was conducted to determine the prevalence of thyroid dysfunction in patients with T2DM attending Mekran Medical College, Turbat, and to assess its impact on glycemic control.

## Materials and methods

A hospital-based cross-sectional study was carried out at Nawaz Sharif Medical College, Aziz Bhatti Shaheed Teaching Hospital, Gujrat, Pakistan, over a six-month period from January to June 2025. The study was planned and reported according to the Strengthening the Reporting of Observational Studies in Epidemiology (STROBE) recommendations [11]. Ethical clearance was granted by the Institutional Ethical Review Committee of Nawaz Sharif Medical College, Aziz Bhatti Shaheed Teaching Hospital (Ref. No. ERC/NMC-HI-ERC/24/1042; approval date: December 13, 2024). Before participation, written informed consent was obtained from every patient.

The required sample size was estimated using the WHO sample size formula. An expected prevalence of thyroid dysfunction of 20% among patients with T2DM was selected based on previous published evidence reporting values between 12% and 23% [12]. Using a 95% confidence level and a 5% margin of error, the minimum sample size was calculated to be 246 participants. To allow for incomplete records, 260 patients were recruited. Participants were selected by consecutive non-probability sampling.

Patients aged 18 years or older with established T2DM for at least six months were considered eligible. Patients diagnosed with type 1 diabetes mellitus or gestational diabetes were not included. Individuals with thyroid cancer, previous thyroid surgery, pregnancy, severe liver or kidney disease, acute infectious illness, or treatment with drugs known to interfere with thyroid function, including amiodarone, lithium, and corticosteroids, were also excluded.

Information regarding age, sex, duration of diabetes, body mass index (BMI), smoking history, blood pressure, and current antidiabetic therapy was collected through direct interviews and review of hospital records. After overnight fasting, venous blood samples were obtained under standard laboratory conditions. Fasting blood glucose and glycated hemoglobin (HbA1c) were measured using routine laboratory procedures. Thyroid status was evaluated by estimating serum thyroid-stimulating hormone (TSH), free triiodothyronine (FT3), and free thyroxine (FT4). Patients were categorized as euthyroid or having overt or subclinical hypo- or hyperthyroidism according to the laboratory reference values used by the hospital.

Factors that could influence the relationship between thyroid dysfunction and glycemic control, including age, sex, BMI, duration of diabetes, smoking, hypertension, and antidiabetic treatment, were recorded and adjusted for during statistical analysis. Records lacking essential laboratory measurements were excluded from the final dataset. However, participants with limited missing demographic information were retained if the primary outcome variables were complete.

Statistical analysis was performed using IBM SPSS Statistics for Windows, Version 26 (Released 2019; IBM Corp., Armonk, New York). Quantitative variables are presented as mean ± standard deviation, whereas qualitative variables are expressed as frequency and percentage. Group differences for continuous variables were assessed using the independent samples t-test or one-way analysis of variance, while categorical variables were compared using the chi-square test. Multivariable logistic regression was used to examine whether thyroid dysfunction was independently associated with poor glycemic control after adjustment for relevant confounding factors. Statistical significance was considered at a two-sided p-value below 0.05.

## Results

During the study period, 260 patients with T2DM were recruited. Eight participants were excluded because thyroid function test results were unavailable, leaving 252 patients for the final analysis. The study population had a mean age of 52.8 ± 10.9 years. Males accounted for 142 (56.3%) participants, while 110 (43.7%) were females. The average duration of diabetes was 8.1 ± 4.7 years, and the overall mean HbA1c was 8.2 ± 1.6%.

Among the analyzed patients, thyroid dysfunction was detected in 68 (27.0%), whereas 184 (73.0%) had normal thyroid function. Subclinical hypothyroidism was the most frequent thyroid disorder observed, with overt hypothyroidism being the second most common diagnosis (Table 1).

**Table 1:** Frequency of thyroid dysfunction among study participants (n=252) The values are reported as frequency (percentage); n(%), using the final study population of 252 participants as the denominator. This table summarizes the distribution of thyroid status only; therefore, no statistical comparison was performed. The thyroid dysfunction group comprised patients with subclinical hypothyroidism, overt hypothyroidism, subclinical hyperthyroidism, and overt hyperthyroidism.

Thyroid Status	n (%)
Euthyroid	184 (73.0)
Subclinical hypothyroidism	38 (15.1)
Overt hypothyroidism	18 (7.1)
Subclinical hyperthyroidism	8 (3.2)
Overt hyperthyroidism	4 (1.6)
Total thyroid dysfunction	68 (27.0)

The demographic and clinical profile of participants according to thyroid status is presented in Table 2. Patients with thyroid dysfunction were more likely to be female, had a longer duration of diabetes, and showed a higher mean BMI than euthyroid participants. These differences were statistically significant.

**Table 2:** Comparison of demographic and clinical characteristics according to thyroid status Continuous variables are expressed as mean ± standard deviation (SD), whereas categorical variables are presented as number (n) and percentage (%). Differences between continuous variables were assessed using the independent samples t-test, while the chi-square test was applied for categorical variables. Statistical significance was defined as a p-value below 0.05. BMI: Body Mass Index.

Variable	Euthyroid (n=184)	Thyroid Dysfunction (n=68)	Test Statistic	p-value
Age (years)	51.9 ± 10.5	55.2 ± 11.6	t = 2.14	0.034
Male, n (%)	112 (60.9)	30 (44.1)	χ² = 5.46	0.019
Female, n (%)	72 (39.1)	38 (55.9)	χ² = 5.46	0.019
Duration of diabetes (years)	7.3 ± 4.1	10.1 ± 5.3	t = 4.23	<0.001
BMI (kg/m²)	27.8 ± 4.3	30.1 ± 4.8	t = 3.34	0.001
Current smokers, n (%)	41 (22.3)	13 (19.1)	χ² = 0.29	0.589
Hypertension, n (%)	94 (51.1)	45 (66.2)	χ² = 4.49	0.034
Insulin use, n (%)	58 (31.5)	31 (45.6)	χ² = 4.27	0.039

Table 3 summarizes the biochemical findings in the euthyroid and thyroid dysfunction groups. Compared with euthyroid participants, patients with thyroid dysfunction had significantly higher fasting blood glucose and HbA1c values, indicating poorer glycemic control.

**Table 3:** Comparison of laboratory parameters according to thyroid status Results are presented as mean ± standard deviation (SD). Differences in laboratory parameters between the euthyroid and thyroid dysfunction groups were evaluated using the independent samples t-test, with a p-value <0.05 considered statistically significant. HbA1c: Glycated Hemoglobin; TSH: Thyroid-Stimulating Hormone; FT3: Free Triiodothyronine; FT4: Free Thyroxine.

Parameter	Euthyroid (n=184)	Thyroid Dysfunction (n=68)	Test Statistic	p-value
Fasting blood glucose (mg/dL)	161.4 ± 42.8	189.7 ± 49.6	t = 4.41	<0.001
HbA1c (%)	7.8 ± 1.4	9.1 ± 1.7	t = 5.99	<0.001
TSH (mIU/L)	2.84 ± 1.12	6.92 ± 4.48	t = 7.31	<0.001
FT3 (pg/mL)	3.21 ± 0.58	2.86 ± 0.71	t = 3.97	<0.001
FT4 (ng/dL)	1.24 ± 0.23	1.01 ± 0.31	t = 6.08	<0.001

As shown in Table 4, impaired glycemic control was more common among patients with thyroid dysfunction. Using an HbA1c threshold of ≥7%, poor glycemic control was identified in 57 (83.8%) patients with thyroid dysfunction compared with 108 (58.7%) euthyroid patients, and this difference was statistically significant (p<0.001).

**Table 4:** Association between thyroid dysfunction and glycemic control Categorical variables are presented as numbers (n) and percentages (%). The association between thyroid status and glycemic control categories was examined using the chi-square test. Statistical significance was set at a p-value of less than 0.05. HbA1c: Glycated Hemoglobin.

Glycemic Control	Euthyroid n (%)	Thyroid Dysfunction n (%)	Test Statistic	p-value
HbA1c <7%	76 (41.3)	11 (16.2)	χ² = 14.67	<0.001
HbA1c ≥7%	108 (58.7)	57 (83.8)		<0.001

To determine factors independently associated with poor glycemic control (HbA1c ≥7%), a multivariable logistic regression model was constructed. After controlling for age, sex, BMI, duration of diabetes, smoking status, hypertension, and insulin use, thyroid dysfunction remained an independent predictor of poor glycemic control, as presented in Table 5.

**Table 5:** Multivariable logistic regression analysis for poor glycemic control (HbA1c ≥7%) Multivariable logistic regression analysis was performed to identify factors independently associated with poor glycemic control (HbA1c ≥7%). Variables entered into the model included age, gender, body mass index, duration of diabetes, smoking status, hypertension, insulin use, and thyroid dysfunction. Results are presented as adjusted odds ratios (ORs) with 95% confidence intervals (CIs). A p-value <0.05 was considered statistically significant. OR: Odds Ratio; CI: Confidence Interval; BMI: Body Mass Index; HbA1c: Glycated Hemoglobin.

Variable	Adjusted OR	95% CI	Wald	p-value
Thyroid dysfunction	2.74	1.41–5.33	8.78	0.003
Age (years)	1.01	0.98–1.04	0.58	0.448
Female gender	1.19	0.68–2.08	0.39	0.533
BMI (kg/m²)	1.08	1.02–1.15	6.45	0.011
Duration of diabetes (years)	1.12	1.05–1.19	12.82	<0.001
Smoking	1.24	0.61–2.51	0.36	0.546
Hypertension	1.38	0.79–2.40	1.28	0.258
Insulin use	1.71	1.01–2.90	3.98	0.046

## Discussion

Thyroid dysfunction is an important problem in patients with T2DM. Both diabetes and thyroid disease affect body metabolism. Poor control of one condition can disturb the other [13]. In the present study, thyroid dysfunction was found in 27.0% of diabetic patients. This shows that thyroid problems are common in patients with T2DM. Similar findings were reported by Khassawneh et al., who found thyroid dysfunction in 26.7% of type 2 diabetic patients [14]. A Pakistani study by Bukhari et al. also reported a high burden of thyroid dysfunction in diabetic patients [15].

Subclinical hypothyroidism was the most common thyroid disorder in our study. This finding is also supported by previous studies. Subclinical hypothyroidism is usually silent. Many patients do not have clear symptoms. This may be the reason that it remains undiagnosed in routine diabetic care. Raised TSH with normal or near-normal FT3 and FT4 shows early thyroid imbalance [16]. This stage is clinically important because it may worsen metabolic control with time.

In our study, thyroid dysfunction was more common in females. This finding is expected. Thyroid disorders are generally more common in women due to hormonal and immune-related differences. Previous studies have also shown female gender as an important factor for thyroid dysfunction in diabetic patients [17]. Older age was also linked with thyroid dysfunction in our results. With increasing age, thyroid gland function may decline [18]. Long duration of diabetes may also disturb endocrine balance and increase the chance of thyroid abnormality [19].

Patients with thyroid dysfunction had a higher BMI. This finding is logical because hypothyroidism can reduce metabolic rate. It may cause weight gain and difficulty in weight control. Obesity can also increase insulin resistance. This can further disturb blood glucose control. Therefore, diabetes, obesity, and thyroid dysfunction may affect each other in the same patient [20].

The duration of diabetes was significantly higher in patients with thyroid dysfunction. This shows that thyroid problems may become more common as diabetes becomes older. Long-standing diabetes is linked with chronic inflammation, insulin resistance, and metabolic stress. These changes may affect thyroid hormone balance. Similar observations have been reported in earlier studies where a longer duration of diabetes was related to thyroid dysfunction [19].

The most important finding of our study was poor glycemic control in patients with thyroid dysfunction. These patients had significantly higher fasting blood glucose and HbA1c levels than euthyroid patients. Similar findings were reported by Elgazar et al., who observed a higher frequency of thyroid dysfunction among diabetic patients with poor glycemic control [21]. Cefalo et al. also found that elevated HbA1c levels were associated with subclinical hypothyroidism in patients with T2DM [22]. These findings support the close relationship between thyroid dysfunction and impaired glycemic control.

The possible reason is that thyroid hormones play an important role in glucose use, insulin secretion, and insulin sensitivity. Hypothyroidism may reduce glucose uptake by tissues and may increase insulin resistance. Hyperthyroidism can increase glucose absorption and hepatic glucose production. Both conditions can disturb blood sugar levels [23]. This explains why patients with thyroid dysfunction showed poorer HbA1c control in our study.

Hypertension and insulin use were also more common in patients with thyroid dysfunction. This may show more advanced metabolic disease in these patients. Patients using insulin usually have longer disease duration or poor glycemic control. Hypertension is also common in long-standing diabetes. These factors may act together and increase the overall metabolic burden [24].

In multivariable analysis, thyroid dysfunction remained independently associated with poor glycemic control after adjustment for age, gender, BMI, duration of diabetes, smoking, hypertension, and insulin use. This suggests that thyroid dysfunction is not only an associated finding. It may have an independent role in poor diabetes control. BMI, duration of diabetes, and insulin use were also significant factors. These findings are clinically understandable because obesity, longer diabetes duration, and insulin requirement are commonly linked with difficult glycemic control [25].

The clinical importance of this study is that thyroid screening may be useful in patients with T2DM, especially in females, obese patients, older patients, and those with poor HbA1c control. Early detection of thyroid dysfunction may help improve diabetic management. It may also reduce future complications.

This study had some limitations. It was a single-center cross-sectional study, so cause and effect cannot be confirmed. Thyroid antibodies were not tested, so autoimmune thyroid disease could not be assessed. Dietary iodine status and drug adherence were also not evaluated. Future multicenter studies with follow-up are needed. Further research should also assess whether treatment of thyroid dysfunction improves HbA1c and diabetic outcomes.

## Conclusions

Early identification of thyroid dysfunction may help clinicians optimize diabetes management and improve metabolic outcomes. Routine thyroid assessment may be particularly beneficial in patients with poor glycemic control or long-standing diabetes. Larger multicenter prospective studies are needed to confirm these findings and evaluate the effect of timely treatment of thyroid dysfunction on long-term diabetic complications.
